# A Spatiotemporal Deep Neural Network Useful for Defect Identification and Reconstruction of Artworks Using Infrared Thermography

**DOI:** 10.3390/s22239361

**Published:** 2022-12-01

**Authors:** Morteza Moradi, Ramin Ghorbani, Stefano Sfarra, David M.J. Tax, Dimitrios Zarouchas

**Affiliations:** 1 Structural Integrity & Composites Group, Aerospace Engineering Faculty, Delft University of Technology, Kluyverweg 1, 2629 HS Delft, The Netherlands; 2 Center of Excellence in Artificial Intelligence for Structures, Prognostics & Health Management, Aerospace Engineering Faculty, Delft University of Technology, Kluyverweg 1, 2629 HS Delft, The Netherlands; 3 Pattern Recognition Laboratory, Faculty of Electrical Engineering, Mathematics and Computer Science, Delft University of Technology, Van Mourik Broekmanweg 6, 2628 XE Delft, The Netherlands; 4 Department of Industrial and Information Engineering and Economics (DIIIE), University of L’Aquila, Piazzale E. Pontieri 1, Monteluco di Roio, 67100 L’Aquila, Italy

**Keywords:** infrared thermography, non-destructive testing, cultural heritage assets, deep learning, spatiotemporal deep neural network

## Abstract

Assessment of cultural heritage assets is now extremely important all around the world. Non-destructive inspection is essential for preserving the integrity of artworks while avoiding the loss of any precious materials that make them up. The use of Infrared Thermography is an interesting concept since surface and subsurface faults can be discovered by utilizing the 3D diffusion inside the object caused by external heat. The primary goal of this research is to detect defects in artworks, which is one of the most important tasks in the restoration of mural paintings. To this end, machine learning and deep learning techniques are effective tools that should be employed properly in accordance with the experiment’s nature and the collected data. Considering both the temporal and spatial perspectives of step-heating thermography, a spatiotemporal deep neural network is developed for defect identification in a mock-up reproducing an artwork. The results are then compared with those of other conventional algorithms, demonstrating that the proposed approach outperforms the others.

## 1. Introduction

The preservation of cultural heritage assets has been nowadays attended because they carry valuable information. Therefore, the use of non-destructive testing (NDT) procedures in conservation is highly valued by restorers and art historians [1]. Thermal non-destructive testing is a smart option to inspect cultural heritage objects since surface and subsurface defects can be detected by exploiting the 3D diffusion inside the object induced by external radiation [2,3]. However, a painting surface is one of the challenging items for infrared thermography (IRT) [4] as an NDT method because the pigments composing the colors cause variations in the emissivity of the surface, which is the most important coefficient in the emitted radiation energy and Stefan–Boltzmann law [5].

External surface treatments, typically given by spray, are frequently employed as a technique to improve the emissivity value in order to increase the thermal contrast resulting from subsurface faults projected on the surface. This strategy, however, is not always appropriate, especially when sensitive layers of items, such as cultural heritage and artworks, must be investigated [6,7]. Therefore, other techniques without any manipulation of the objects should be considered to identify the defects of an artwork.

Data analysis by taking the heat equations into account can be helpful in extracting valuable knowledge from thermograms recorded by an IR camera [8,9]. Yet, because an artwork may have complex damage configurations (various sizes, depths, materials, and types), analyzing IR data using only physical models is extremely difficult, especially when access to thermal data is limited to the external surface. Data-driven models and artificial intelligence (AI) are key mathematical approaches to overcoming this challenge.

In recent years, infrared machine vision has gained increasing interest in various domains thanks to the increasing growth of machine learning, notably with deep learning (DL) algorithms that use multiple layer networks to extract higher-level features from raw IR input sequentially [10]. The complexity of identifying damage in an artwork can be addressed by employing AI, particularly DL. Despite significant research advances in IRT processing using unsupervised learning, generally employed detection algorithms still have difficulties in defect identification due to weak signal-to-noise ratio (SNR), complicated interference, and so on. The development of supervised learning to research IRT is a prospective trend based on the spatial-temporal physical properties of the IRT sequences [11]. To this end, thermal video can be analyzed from two perspectives:

1. The aspect of temporal information that includes the temperature variation of each pixel over time and could be regarded as a time-series input;
2. The spatial information aspect, which includes the temperature variation of each frame (at one moment) over all pixels, could be regarded as an image input.

In the present work, a deep neural network (DNN) framework is designed to classify pixels into healthy and defective regions, presenting the pertinent intact and damaged areas of the object under inspection. The proposed framework consists of two sub-models: a multilayer perceptron (MLP) to classify each time-series (1D signal) into healthy or defective pixels, and a convolutional neural network (U-Net) to segment images into healthy or defective areas. These two networks are fused sequentially together in order to enhance performance in such a way that after training the former one, the latter is trained. The developed framework’s performance is compared to the results of popular algorithms such as pulsed phase thermography (PPT), principal component thermography (PCT), and thermographic signal reconstruction (TSR).

The remaining sections of the paper are structured as follows: Section 2 summarizes and discusses the history of IRT in cultural heritage and IRT using AI. After that, Section 3 will explain the experimental setup. The methodology, including spatial and temporal networks, will be addressed in Section 4. After that, Section 5 will describe the results and discussion. Finally, the paper is concluded in Section 6, which also provides an outlook on future work. 

## 2. Background on IRT in Cultural Heritage and IRT Using AI

IRT is widely used in a variety of industries, from the aviation industry [12] to civil engineering [13], from stress analysis [14] to damage characterization [15], from diagnostics [16] to prognostics [17]. Owing to its advantages, this NDT method is also one of the most beneficial inspections for cultural heritage assets, a topic that is gaining more and more attention and progress [18]. For instance, F.J. Madruga et al. [19] applied step-heating (SH) thermography and preliminary speckle patterns to explore the impact of wood’s degree of humidity, which is crucial for its construction and restoration of artworks. Contrary to some other areas of investigation, the examination of historical items demands extra care with regard to safety. For example, the investigation of the cast-iron Buddha head created in China during the Song Dynasty (AD 960-1279) is a very delicate and important process that was carried out by IRT [20].

Raw thermal images should be processed since they are ineffective for identifying subsurface defects in cultural heritage assets. Different post-processing techniques, such as PCT, PPT, TSR, thermal signal area (TSA) [21], 1D, 2D, and 3D wavelet analysis [22], can be employed to process and interpret thermograms. In this regard, measurements of the depth and size of flaws in test specimen paintings on wooden panels have been done quantitatively. C. Ibarra-Castanedo et al. [23] used pulsed thermography (PT) processing techniques, such as differential absolute contrast (DAC) and PPT, to diagnose hidden faults, which showed interesting quantitative results. Pulse-compression thermography and hyper-spectral imaging were coupled by S. Laureti et al. [24] to inspect fabricated and non-fabricated targets in a canvas painting mockup. The findings showed that for both applied approaches, PCT and independent component thermography (ICT) maximized the number of targets retrieved during the post-acquisition steps. A factor analysis thermography (FAT) technique was adopted by K. Liu et al. [25] to automatically extract fault features from thermal images captured from panel paintings. A fuzzy c-means (FCM) image segmentation algorithm was applied to factor-loaded images to diminish background interference on human visual identification. In comparison to PCT, the results demonstrated the proposed method’s effectiveness. Although several studies have been conducted to develop the IRT processing algorithms, more precision is still required, and there is still a shortage of automation in the IRT data interpretation.

In this regard, DL is a data processor with a flexible option for increasingly automated assessment [26]. Z. Wei et al. [27] developed a U-Net-based model to predict the damage area for the impacted curve-shaped CFRP specimens inspected by IRT. They employed PCT to capture the ground truth maps needed for model training. It should be noted that the homogeneous and less reflective colors that cover the surface of the CFRP specimens make damage detection for them easier than panel painting for IRT. For various infrastructures (including buildings, historical landmarks, and civil infrastructures), thermograms obtained were processed using the mask region-convolution neural network (Mask R-CNN) in conjunction with an automatic thermogram preprocessing algorithm with a focus on water-related issues and thermal bridges [28]. For the purpose of diagnosing breast cancer, a deep convolutional neural network (CNN) including transfer learning has been developed to automatically classify thermograms into two categories: normal and abnormal [29]. Although more samples can enhance the training process of the DL models for some of the applications stated above, sufficient samples can still be supplied in comparable conditions and made of similar materials in order to acceptably train the models. However, in the field of cultural heritage, only one specimen is available, notably for paintings which have a particular fabrication as well as pigment pattern and materials. Furthermore, because of the variation in emissivity throughout the canvas’ surface, the pigment variance over its surface causes erroneous recorded thermal maps [4].

To address the above-mentioned challenges, it is beneficial to analyze the thermal data from various aspects in order to discover the spatial relationships among adjacent pixels and the temporal relationships between successive frames. In the current work, an MLP optimized via scaled conjugate gradient backpropagation is implemented to analyze temporal information, and a CNN (U-Net) with an Adam optimizer is applied to evaluate spatial information. Together, these components make up a spatiotemporal deep neural network (STDNN).

## 3. Experimental Setup

**Figure 1:**
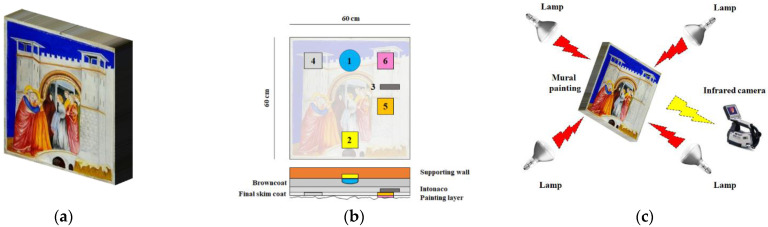
(**a**) Mock-up photograph, (**b**) defects map (detachment between the wall and the browncoat: void (1), moisture into the masonry: sponge soaked in water (2), detachment between the intonaco and the final skim coat: void (3), efflorescences: salts (4), cracks: sand (5), and flaking: poly-acetovinyl (6)), and (**c**) sketch of the experimental IRT setup.

The IRT-inspected artwork is a replica of Giotto’s “Meeting at the Golden Gate” (a mural painting) that is preserved in Padua’s Scrovegni Chapel (Italy). The size of the replica is 60 × 60 cm. The sample contains several faults at various layers, indicating typical degrading mural painting faults. A photograph of the replica, a map of defects, and a sketch of the experimental setup for the laboratory IRT inspection can be seen in Figure 1. Details about the different fabricated defects and more information have been provided in [30].

The sample was stimulated by four halogen lamps (OSRAM SICCATHERM, 250 W), and the thermal response of the surface was recorded by an infrared camera (FLIR S65 HS, 7.5–13 μm, 320 × 240, 50 Hz). The heating and cooling phases lasted 52.5 and 164 s, respectively (for a total of around 216.5 s). The final thermography dataset contains 10826 thermal images, of which the frames with a 25 Hz rate will be collected for further analysis to reduce processing costs, and the total number of final frames is 434. In addition, each image is cropped to remove the additional marginal pixels, reducing the size of each frame to 230 × 230. As a result, the network’s input data set size is 230 × 230 × 434.

## 4. Methodology

**Figure 2:**
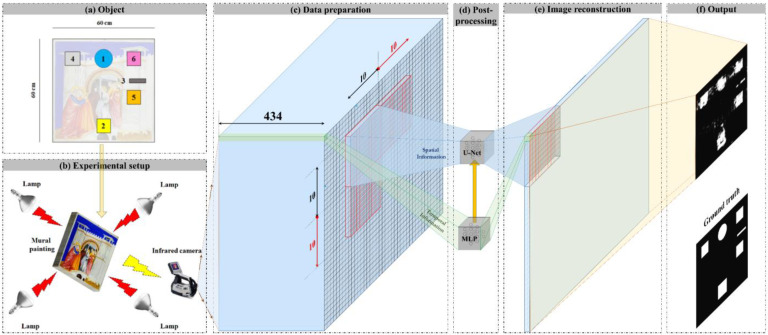
The complete flowchart of the current work includes: (**a**) object preparation for inspection, (**b**) configuration of the experimental set-up, (**c**) data preparation, (**d**) post-processing algorithm, (**e**) image reconstruction, and (**f**) output.

**Figure 3:**
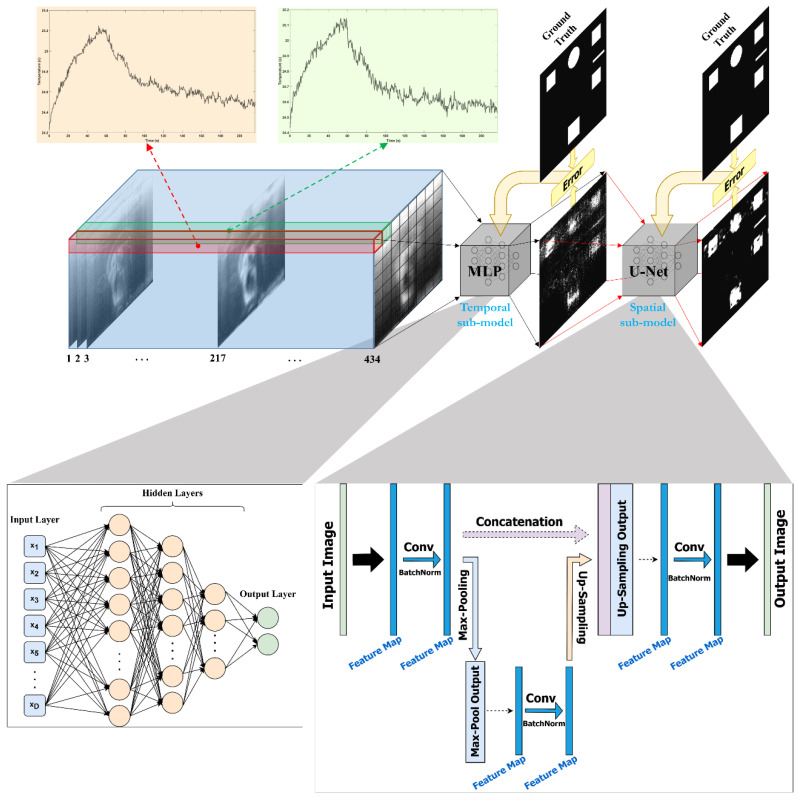
The proposed spatiotemporal deep neural network (STDNN). First, the temporal signals for each pixel over time are classified using the MLP model, whose outputs are then segmented and spatially processed using the U-Net model.

The complete flowchart of the present work, including all steps taken, is shown in Figure 2. This section outlines the data preparation, the proposed framework (STDNN, shown in Figure 3), and evaluation metrics. The framework is divided into two learning steps using different sub-models: temporal and spatial sub-models. In the first step, an MLP is used to classify the temporal signals related to each pixel of the input image as healthy or defective. In the next step, the output of the MLP model is used as the input of a U-Net model to segment images into healthy and faulty regions based on the relationships between surrounding pixels in spatial information. The U-Net model outputs, including training and test sets, are concatenated to reconstruct the full final image.

### 4.1. Data Preparation

In order to make the input data ready for the first step of the proposed framework, the thermal signals relevant to each pixel are labeled using the ground truth image, which was built artificially using the actual locations and dimensions of the defects. As can be seen in Figure 2c, the dataset is then windowed across the spatial point of view by a mask with a size of 10-by-10 pixels, which is a patch of neighboring pixels, and the moving stride is 10 pixels in both directions of the image’s height and width (i.e., zero overlap). As a result, the dataset is composed of 23 × 23 patches of 3-dimensional data, each of which is 10 × 10 × 434 pixels in size. This type of partitioning was carried out because the dataset needed to be divided into training and test datasets from only one specimen, and pixels near to each other in the spatial network needed to be imported into the sub-model for the segmentation task and morphology based on topology. The dataset, including 529 (23 × 23) patches, is divided into training and test datasets with a ratio of 7:3. As a result, the training and test datasets are composed of 370 and 159 patches, respectively, with each patch size of 10 × 10 × 434. Due to the randomness in making the training and test sets, the data has been split ten times to have different datasets for fair experimental results.

### 4.2. Temporal Network

For the first step of the proposed framework, each thermal signal related to a pixel from the patches is imported separately into the MLP model to be classified as healthy or defective. An MLP model is used, comprising three hidden layers, each containing 20, 10, and 5 neurons, respectively. The hyperbolic tangent (tan-sigmoid) function is used as the activation function in all hidden layers, whereas the logistic (log-sigmoid) function is used in the output layer. The scaled conjugate gradient (SCG) backpropagation developed by M. Moller [31] is adopted as the optimizer, which is dependent on conjugate directions. In contrast to other conjugate gradient techniques, SCG does not conduct a linear search with each iteration which increases the computational cost. The time-consuming line search was avoided when developing the SCG. The SCG algorithm is more reliable and independent of user-defined parameters because the learning rate is a function of the quadratic approximation of the error function. A binary cross-entropy function with a regularization parameter of 0.1 is used as the loss function. Note that training runs for 1000 epochs. In order to assess the randomness of the deep learning model settings for the MLP model, the training process is repeated ten times. Then, the output of the first repeat of the training process from each dataset has been selected as the input of the U-Net model in the next step of the proposed framework. The outputs of the MLP model, with the order of initial patches, create the input for the next step of the proposed framework.

### 4.3. Spatial Network

For the second step, a U-Net model [32] is employed to segment images into healthy and faulty regions based on the relationships between surrounding pixels in the spatial information. The special architecture of the used U-Net model consists of convolution layers followed by an exponential linear unit (ELU) activation function, batch normalization, and MaxPooling layers. From a different perspective, this architecture is composed of an encoder network followed by a decoder one. U-Net, as opposed to a simple autoencoder architecture, has additional interconnections between the encoder and decoder sections. The details of the implemented U-Net model’s architecture are presented in Table 1. The default settings of the Keras API are used for the parameters that are not mentioned. The Adam optimizer with a learning rate of 0.001 and a decay rate of 0.0001 is used. The batch size is 8, and training runs for 300 epochs. Moreover, binary cross-entropy is used as the loss function. Similar to the spatial network, the training process is repeated ten times to assess the randomness of the deep learning model settings. Patches of 10 × 10 × 434 are inputted to the U-Net, and then all resultant patches of 10 × 10 × 1, including training and test ones, are concatenated to reconstruct the full image of 230 × 230.

### 4.4. Evaluation Metrics

Due to the class-imbalanced situation in our experiments, the performance of the proposed framework is evaluated using different evaluation methods such as Accuracy, Recall (sensitivity), Precision, F1-score, and AUC, which is the area under the ROC (receiver operating characteristic) curve:

(1) $$Accuracy=\frac{TP+TN}{TP+FP+TN+FN}$$

(2) $$Recall=\frac{TP}{TP+FN}$$

(3) $$Precision=\frac{TP}{TP+FP}$$

(4) $$F1-score=\frac{2\times Precision\times Recall}{Precision+Recall}$$

where FP and FN stand for the number of false positive and negative predictions, while TP and TN stand for the number of true positive and true negative predictions, respectively. Macro-Averaged is used to calculate the Recall, Precision, and F1-Score metrics.

## 5. Results and Discussion

The averaged AUC over ten repeats across ten datasets for the MLP is presented in Table 2. The overall mean AUC performance of the MLP shows that this model is capable of classifying the pixels into healthy or defective since it has a mean AUC performance of 0.88 with a very low standard deviation over ten different test sets. Moreover, the low standard deviation in the performance of the MLP sub-model over ten repeats for each dataset shows the stability of the model with different weight initializations.

**Figure 4:**
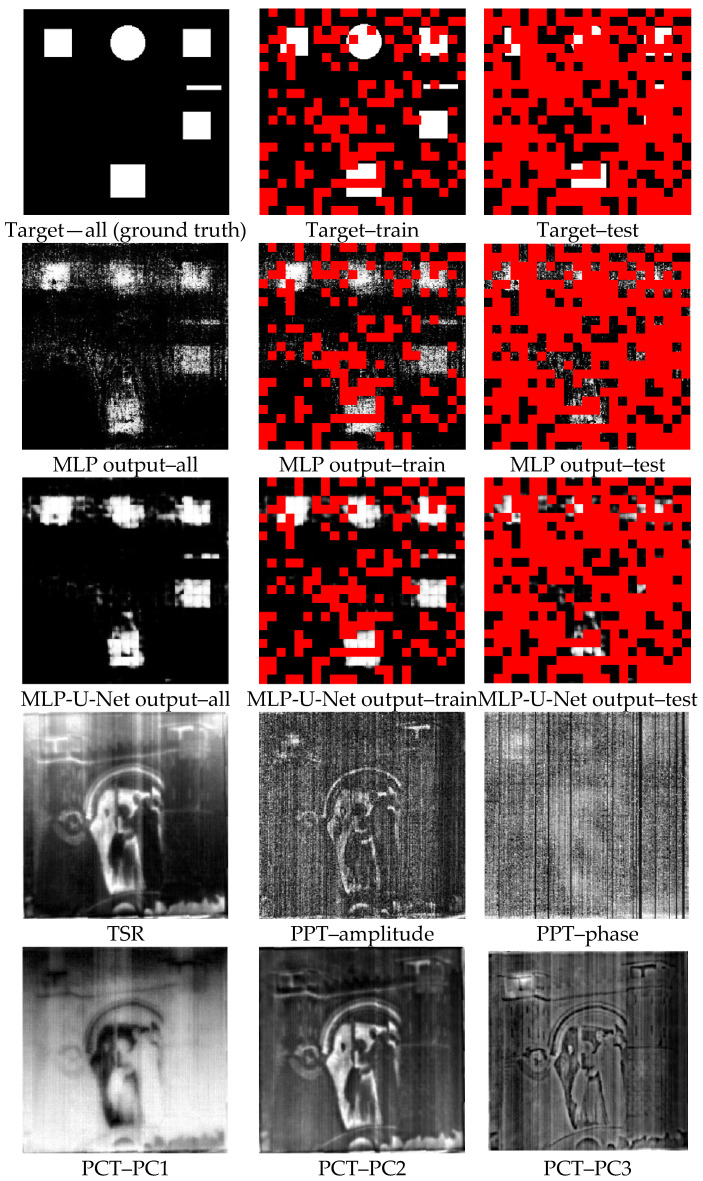
Results of MLP based on only temporal information (the 2nd row), MLP-U-Net based on both temporal and spatial information (the 3rd row), and the well-known algorithms of TSR, PPT, and PCT (the 4th and 5th rows) compared to the ground truth image (the 1st row) for the first dataset. The red color for the first three rows represents the unused pixels for training or test.

The output images obtained by the temporal sub-model (MLP) from the first dataset and the first repeat can be seen in Figure 4 (the 2nd line).

Table 3 indicates the U-Net model’s performance across all ten datasets using four evaluation metrics. Based on the overall mean AUC performance results, the U-Net sub-model can segment images into healthy and faulty regions. The mean AUC performance of the U-Net model over all ten test sets is 0.94, with a low level of variability, which proves the stability and good performance of the model. Moreover, the performance of other evaluation metrics demonstrates the capability of the U-Net sub-model in this task. Precision estimates the validity for the minority class in the case of a class-imbalanced situation since it is a measure that quantifies the amount of correct positive (minority class, which refers to the defective pixels in the current work) predictions. The results indicate that the mean Precision on the test set is 0.84 with a low standard deviation, which can be considered an outstanding performance. Although Precision is valuable and the findings appear to be excellent, it does not reflect how many true positive class samples are predicted as belonging to the negative class (majority class, which refers to the healthy pixels in the current work). Precision only indicates the correct positive prediction out of all positive predictions, but Recall metric, as opposed to Precision, shows missed positive predictions. The Recall is a measure that reflects the number of correct positive predictions made out of all possible positive forecasts. The purpose in the case of an imbalanced dataset is to optimize Recall without diminishing Precision. Nevertheless, both criteria are often in contradiction, as increases in Recall often come at the expense of declines in Precision. The F1-score integrates Precision and Recall into a single metric that encompasses both. The overall F1-score of the U-net model on the test set demonstrates that the model performs acceptably in segmenting the image into healthy or faulty regions based on the spatial information relationships between surrounding pixels. Figure 4 (the 3rd line) depicts the images generated by the spatial sub-model (U-Net) once it was performed on the MLP outputs for the first dataset and first repeat.

The TSR results and the amplitude and phase of PPT at the frame with the high contrast (maximum kurtosis) are displayed in Figure 4 (the 4th line) to be compared with the proposed framework. As can be seen, only a shadow of defect No. 6, which is relatively large and shallow (according to Figure 1), may be distinguishable by TSR. The misleading effect of the emissivity of pigments is obvious in TSR results. 

On the other hand, the PPT algorithm, which is based on the Fourier transform, performs better in smoothing the emissivity problem; however, detectability is not acceptable. Defect No. 4 may be assumed to be discovered in the amplitude of PPT. A slight shadow of defects No. 4 and No. 1 may be seen in the PPT phase. These suspect detections are primarily due to prior knowledge of the locations and dimensions of the defects. However, in some cases, this is not practical in reality.

Figure 4 (the 5th line) depicts the first three principal components (PCs) obtained by PCT. The results show that the PCT algorithm suffers from the emissivity’s deceptive effect. PCs 1 and 2 may include a shadow of defect No. 6. On the other hand, the PC3 seems to be the best one among other PCs, demonstrating the existence of defect No. 4.

Figure 4 illustrates that while the MLP could detect all defects, it was still affected by sparsity and noise among pixels. Taking spatial information into consideration, the U-Net could effectively minimize sparsity and noise in the MLP output. As a result, the proposed model outperforms all reported conventional algorithms in the current work.

The advantages of the conventional methods are that they are simple and straightforward, with few unknown variables that can be determined using deterministic optimization solutions. In other words, they do not have the numerous unknown variables needed to be trained, such as a DNN. However, all phenomena (such as the IRT of an artwork made of non-homogenous material) cannot be parametrized by simple models, which are mainly based on only linear relationships among inputs. This can also be confirmed by the results shown in Figure 4. To cope with this disadvantage of the conventional algorithms, a model based on deep learning was developed, which takes into account both temporal and spatial information. As the results showed, the accuracy of the STDNN is very high and acceptable. The proposed framework was able to rectify the emissivity problem induced by pigment effects. However, its shortcoming is that it is supervised and requires training data from similar types of samples, which is not the case in this work.

## 6. Conclusions

In this work, a spatiotemporal deep neural network (STDNN) was utilized for defect identification in a mock-up reproducing an artwork, taking into account both the temporal and spatial perspectives of SH thermography. Initial results indicated that the mean F1-score evaluation metric is acceptable with a low standard deviation, which can be considered an outstanding performance despite the fact that there is a class-imbalance problem in the data. These results were supported by the AUC scores verifying that the model’s performance was excellent and, more interestingly, stable. Finally, the outcomes of the STDNN were compared to those of other conventional algorithms (i.e., PCT, PPT, TSR). It was found that their results cannot be considered comparable to the MLP-U-Net’s; for example, the effect and reflection of the drawing on the surface are still evident.

It is possible to say that the proposed framework was able to rectify the emissivity problem induced by pigment effects. In the future, training data from similar types of samples (e.g., panel paintings) will be collected in order to reduce the shortcoming of the proposed STDNN mainly linked to the supervised learning approach.

## Figures and Tables

**Table 1:** The U-Net (spatial sub-model) model’s architecture.

Layer Number	Layer Type	Input of Layer
1	Conv2D (Filters: 128, $\left(5\times 5\right)$, Activation Function: ELU, Kernel_Initializer: ‘he_normal’, padding = ‘same’)	MLP’s output
2	Batch Normalization	Layer 1
3	Conv2D (Filters: 128, $\left(3\times 3\right)$, Activation Function: ELU, Kernel_Initializer: ‘he_normal’, padding = ‘same’)	Layer 2
4	Max-Pooling2D (Size: $\left(2\times 2\right)$)	Layer 3
5	Conv2D (Filters: 128, $\left(3\times 3\right)$, Activation Function: ELU, Kernel_Initializer: ‘he_normal’, padding = ‘same’)	Layer 4
6	Batch Normalization	Layer 5
7	Conv2D (Filters: 128, $\left(3\times 3\right)$, Activation Function: ELU, Kernel_Initializer: ‘he_normal’, padding = ‘same’)	Layer 6
8	Conv2D-Transpose (Filters: 128, $\left(3\times 3\right)$$,\;\mathrm{Strides}=\left(2\times 2\right)$, Activation Function: ELU, padding = ‘same’)	Layer 7
9	Conv2D (Filters: 128, $\left(3\times 3\right)$, Activation Function: ELU, Kernel_Initializer: ‘he_normal’, padding = ‘same’)	Concatenation of Layers 3 and 8
10	Batch Normalization	Layer 9
11	Conv2D (Filters: 128, $\left(5\times 5\right)$, Activation Function: ELU, Kernel_Initializer: ‘he_normal’, padding = ‘same’)	Layer 10
12	Dropout (0.5)	Layer 11
Final Layer	Conv2D (Filters: 1, $\left(1\times 1\right)$, Activation Function: Sigmoid)	Layer 12

**Table 2:** AUC calculated from the (temporal sub-model) MLP’s results for the ten generated datasets.

Dataset	1	2	3	4	5	6	7	8	9	10	Mean ± Std_(Mean)_
Training	0.96 ± 0.01	0.96 ± 0.01	0.96 ± 0.01	0.96 ± 0.01	0.97 ± 0.01	0.97 ± 0.00	0.97 ± 0.00	0.96 ± 0.00	0.96 ± 0.01	0.96 ± 0.01	0.96 ± 0.00
Test	0.86 ± 0.01	0.89 ± 0.01	0.87 ± 0.01	0.88 ± 0.01	0.88 ± 0.00	0.87 ± 0.01	0.89 ± 0.01	0.87 ± 0.01	0.89 ± 0.01	0.86 ± 0.01	0.88 ± 0.01

**Table 3:** AUC, Precision, Recall, and F1-Score calculated from the (spatial sub-model) U-Net’s results for the ten generated datasets.

Dataset	AUC		Precision (Specificity)		Recall (Sensitivity)		F1-Score	
	Train	Test	Train	Test	Train	Test	Train	Test
1	1.00 ± 0.00	0.94 ± 0.00	0.97 ± 0.01	0.82 ± 0.03	0.96 ± 0.02	0.75 ± 0.03	0.96 ± 0.01	0.78 ± 0.01
2	1.00 ± 0.00	0.95 ± 0.01	0.91 ± 0.02	0.88 ± 0.02	0.97 ± 0.01	0.85 ± 0.02	0.93 ± 0.01	0.86 ± 0.01
3	1.00 ± 0.00	0.94 ± 0.00	0.95 ± 0.02	0.83 ± 0.04	0.94 ± 0.01	0.78 ± 0.03	0.95 ± 0.01	0.80 ± 0.01
4	0.99 ± 0.00	0.93 ± 0.01	0.92 ± 0.02	0.83 ± 0.01	0.95 ± 0.01	0.79 ± 0.03	0.93 ± 0.01	0.80 ± 0.02
5	0.99 ± 0.00	0.95 ± 0.00	0.95 ± 0.01	0.85 ± 0.02	0.94 ± 0.02	0.81 ± 0.02	0.94 ± 0.01	0.82 ± 0.01
6	1.00 ± 0.00	0.94 ± 0.00	0.89 ± 0.03	0.83 ± 0.02	0.97 ± 0.01	0.81 ± 0.02	0.93 ± 0.02	0.82 ± 0.01
7	1.00 ± 0.00	0.95 ± 0.00	0.96 ± 0.02	0.89 ± 0.03	0.96 ± 0.01	0.83 ± 0.03	0.96 ± 0.01	0.85 ± 0.01
8	0.99 ± 0.00	0.93 ± 0.00	0.92 ± 0.01	0.83 ± 0.02	0.90 ± 0.04	0.74 ± 0.05	0.91 ± 0.02	0.77 ± 0.03
9	1.00 ± 0.00	0.95 ± 0.00	0.96 ± 0.01	0.87 ± 0.02	0.94 ± 0.02	0.81 ± 0.01	0.95 ± 0.01	0.84 ± 0.01
10	1.00 ± 0.00	0.93 ± 0.00	0.92 ± 0.02	0.81 ± 0.02	0.95 ± 0.02	0.78 ± 0.03	0.94 ± 0.01	0.79 ± 0.01
**Mean ± std_(Mean)_**	1.00 ± 0.00	0.94 ± 0.01	0.94 ± 0.02	0.84 ± 0.03	0.95 ± 0.02	0.80 ± 0.03	0.94 ± 0.01	0.81 ± 0.03

## Data Availability

Not applicable.
